# Apolipoprotein E: Isoform Specific Differences in Tertiary Structure and Interaction with Amyloid-β in Human Alzheimer Brain

**DOI:** 10.1371/journal.pone.0014586

**Published:** 2011-01-31

**Authors:** Phillip B. Jones, Kenneth W. Adams, Anete Rozkalne, Tara L. Spires-Jones, Tammy T. Hshieh, Tadafumi Hashimoto, Christine A. F. von Armin, Mathew Mielke, Brian J. Bacskai, Bradley T. Hyman

**Affiliations:** 1 Harvard Medical School, MassGeneral Institute for Neurodegenerative Disease, Massachusetts General Hospital, Charlestown, Massachusetts, United States of America; 2 Klinik für Neurologie, Universitätsklinikum Ulm, Ulm, Germany; Mental Health Research Institute of Victoria, Australia

## Abstract

We applied a novel application of FLIM-FRET to *in situ* measurement and quantification of protein interactions to explore isoform specific differences in Aβ-ApoE interaction and ApoE tertiary conformation in senile plaques in human Alzheimer brain. ApoE3 interacts more closely with Aβ than ApoE4, but a greater proportion of Aβ molecules within plaques are decorated with ApoE4 than ApoE3, lending strong support to the hypothesis that isoform specific differences in ApoE are linked with Aβ deposition. We found an increased number of ApoE N-terminal fragments in ApoE4 plaques, consistent with the observation that ApoE4 is more easily cleaved than ApoE3. In addition, we measured a small but significant isoform specific difference in ApoE domain interaction. Based on our *in situ* data, supported by traditional biochemical data, we propose a pathway by which isoform specific conformational differences increase the level of cleavage at the hinge region of ApoE4, leading to a loss of ApoE function to mediate clearance of Aβ and thereby increase the risk of AD for carriers of the APOEε4 allele.

## Introduction

Inheritance of the Apolipoprotein E (APOE) ε4 allele is the strongest known genetic risk factor for late onset Alzheimer disease (AD). Compared to the more common APOEε3 genotype, homozygous APOEε4 carriers have a ∼10 fold increased risk [1], [2], [3]. Despite the fact that APOEε3 has an allele frequency of 70–80% compared to only 15–20% for APOEε4 [4], approximately 40–65% of AD patients have at least one copy of APOEε4 [5], [6].

Apolipoprotein E (ApoE) is a polymorphic protein with 299 residues (M_r_ = 34000) [7]. The three common isoforms ApoE2, E3, and E4 differ by only two amino acid residues at positions 112 and 158 [8]. ApoE3 and ApoE4 associate with different lipid particles in plasma and appear to adopt different tertiary structures as a result of an Arg61-Glu255 salt bridge, which is altered by the presence of Arg112 in ApoE4. There are two major functional domains, the N-terminal domain (NT), contains the major receptor binding region, the C-terminal domain (CT), contains the lipid binding region, which is also thought to bind Aβ [9], [10]. The two are connected by a flexible hinge region.

The mechanism by which ApoE isoform affects risk of AD is uncertain, with roles proposed in all three of the major pathological hallmarks: Cell death [11], neurofibrillary tangles [12], [13] and senile plaques [3], [14]. Studies *in vivo* show that ApoE4 is associated with increased amyloid deposition in the brain [14], [15], and ApoE protein decorates senile plaques [16]. *In vitro* experiments have shown that there are isoform specific differential interactions of ApoE with Aβ, [17], [18], [19] but it is uncertain whether this is true in the brain because 1. senile plaque structure likely differs from synthetic Aβ fibrils and 2. the lipidation status of ApoE associated with plaques, and possible post-translational modifications including cleavage, are difficult to model *in vitro*. Computation biophysics work recently published by the Paralvrez-Marin group in Sweden [20] has shown that ApoE4 is expected to have a pathological stable intermediary conformation that is mediated by the inter-domain interaction. In addition, there is evidence that ApoE4 is more susceptible to proteolytic cleavage than ApoE3 [21].

The hypothesis that ApoE isoforms adopt different structures in the context of AD pathology therefore remains open (for reviews; see [22], [23], [24]). A potentially useful method for such measurements would be fluorescence lifetime imaging microscopy (FLIM) [25], making use of Förster resonance energy transfer (FRET) [26] to measure the closeness of two protein epitopes. FLIM-FRET makes use of the characteristic fluorescent decay profile of a fluorophore, in particular, its lifetime (τ). When a higher energy (more blue) flourophore (donor) is placed in very close proximity (∼1–10 nm) to a lower energy (more red) fluorophore (acceptor) whereby the emission profile of the donor overlaps with the excitation profile of the acceptor, the donor fluorophore will lose energy to the acceptor resulting in a dimming and shortening of lifetime. By analyzing the time-dependent decay profile of the donor with ultra-high temporal resolution (≈80ps), it is possible to calculate both the percentage of donor molecules that are interacting with acceptors and the distance between the interacting molecules. Until recently, this technique has proved challenging for *in situ* measurements due to contamination of the signal with background tissue autofluorescence. In order to perform FLIM-FRET measurements *in situ*, we have previously developed a suite of analysis techniques [27] called χ^2^ filtering and multiple Gaussian fitting for lifetime evaluation (MUGLE). The consideration of interacting fraction and FRET efficiency as separate parameters is vital to the interpretation of FLIM data, especially with regards to immunohistochemical stains. FRET efficiency is not subject to confounds of differences in labeling efficiency, provided interacting fraction is considered separately.

We reasoned that if ApoE isoforms differentially interact with Aβ in senile plaques, we may be able to detect, using advanced imaging techniques that are sensitive to protein conformation, distinct conformational patterns of ApoE3 and ApoE4, when associated with senile plaques. These analyses found both differences in conformation between ApoE3 and ApoE4, and also evidence for differences in post-translational modification (cleavage) between ApoE3 and ApoE4 protein associated with plaques, which were confirmed with standard biochemical analyses.

## Results

**Figure 1:**
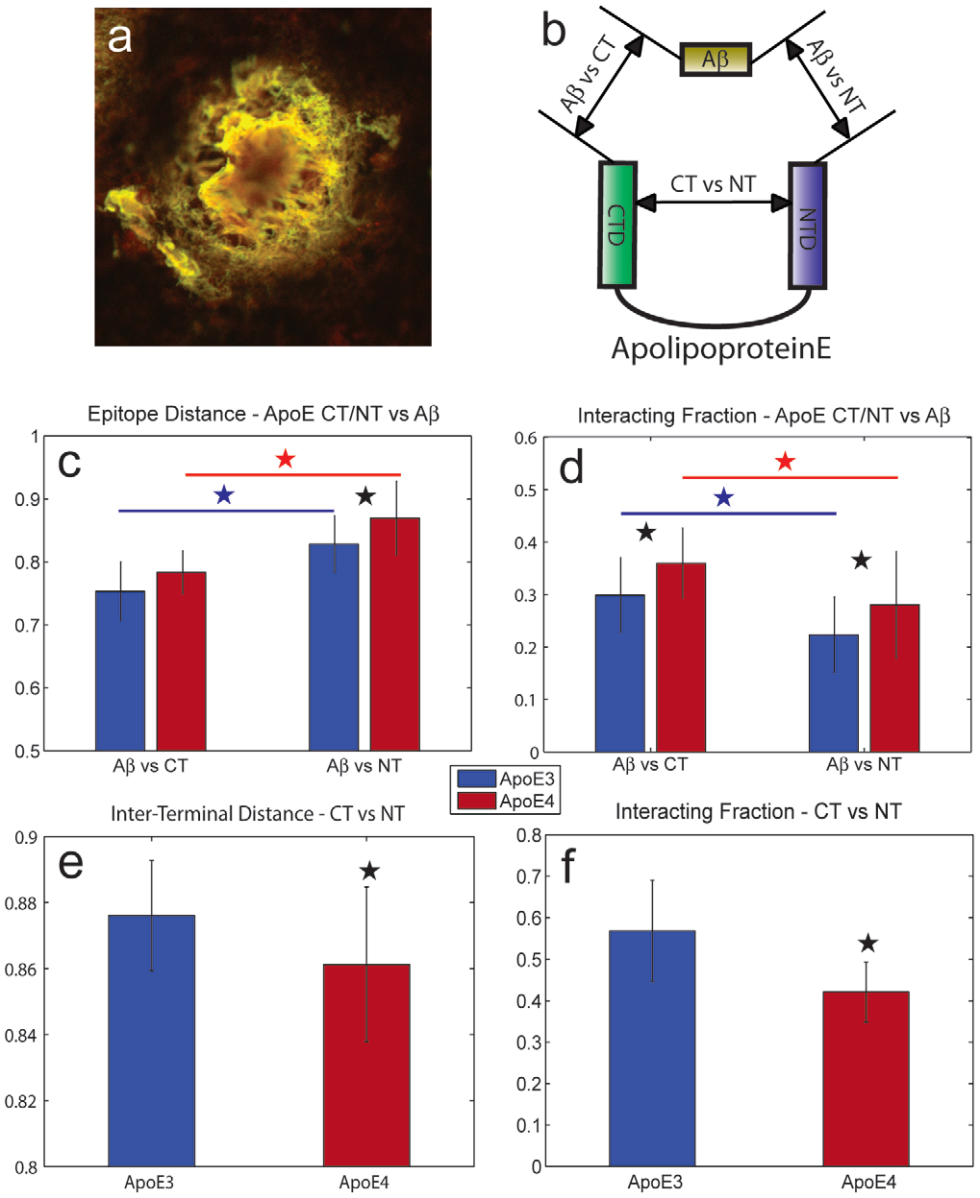
FLIM-FRET study of ApoE conformation and Aβ-ApoE interaction reveals multiple aspects of ApoE4 associated plaque pathology. Inter-epitope distances are normalized to the Förster radius. a) A dense core senile plaque from the cortex of a patient homozygous for ApoEε3. Aβ (green) and ApoE NT (red) are extremely well co-localized which illustrates that the plaque is decorated with ApoE. b) Schematic showing the three FLIM-FRET measurements that were made. We independently measured the interacting fraction and distance between Aβ and both ApoE terminal domains as well between the two ApoE domains. c) ApoE CT is in closer apposition to Aβ than ApoE NT, consistent with the assumption that the hydrophobic lipid binding region interacts with Aβ. The difference in distance is small enough to suggest that ApoE envelops Aβ in a similar fashion than it is known to interact with lipids. d) A significantly greater proportion of Aβ is bound to ApoE in the case of ApoE4. The data suggest a reduced capacity of ApoE4 to induce clearance of Aβ. e) ApoE4 has a slightly tighter terminal interaction. This is surprising because a large difference in inter-terminal interaction is expected from the *in vitro* data. f) ApoE shows a significantly lower numbers of interacting terminal domains. These data are proof that ApoE4 undergoes a greater amount of cleavage either before or after binding to Aβ. Differential cleavage may mediate Aβ clearance or deposition.

We conducted a series of FLIM-FRET experiments using human postmortem tissue sections obtained from the Massachusetts Alzheimer Disease Research Center (ADRC) brain bank (table 1) to examine ApoE conformation when it is associated with senile plaques *in situ* in the Alzheimer brain (figure 1). The sections from individuals were homozygous for either APOEε3 or APOEε4 genotype and all had a diagnoses of AD confirmed at autopsy. Initially, Aβ was immunolabeled with the donor fluorophore (A488) using the Aβ specific antibody (3D6), and either ApoE CT or ApoE NT were immunolabeled with Cy3 using terminal specific antibodies, 3H1 and 6C5 respectively.

**Table 1:** List of cases used for the FLIM-FRET study.

case #	APOE genotype	age at death	Sex	PMI (hrs)	ApoE NT, Aβ	ApoE CT, Aβ	ApoE CT, NT
1	ε3/3	88	F	12	✓		
2	ε3/3	83	F		✓		✓
3	ε3/3	75	F		✓		✓
4	ε3/3	88	M	<12	✓		✓
5	ε3/3	87	F	4		✓	
6	ε3/3	82	M	9		✓	
7	ε3/3	73	F			✓	
8	ε3/3	82	F	7		✓	
Total number of plaques					20	20	14
9	ε4/4	78	F		✓	✓	✓
10	ε4/4	74	M	18	✓		✓
11	ε4/4	78	M	16	✓	✓	
12	ε4/4	68	F	23	✓		
13	ε4/4	81	M			✓	
14	ε4/4	84	F			✓	
15	ε4/4	84	M	24		✓	
16	ε4/4	80	F	10			✓
17	ε4/4						✓
Total number of plaques					19	25	22

The genotype, age of the patient at death, sex and postmortem interval (PMI) is given where available from ADRC records. The check marks show which of the three experiments in which the brain was used. Some brains were used in more than one comparison depending on availability of tissue. For each of the 6 comparisons, the total number of plaques imaged (‘n’) is also given.

To determine the effect to which the proteins were colocalized, two channel confocal images (figure 1a) were acquired and a correlation coefficient was calculated based on the pixel wise correlation of the green and red channels. For all images, strong correlations were observed (R^2^>0.9, p<1e-6), but no difference was observed in the extent of co-localization of ApoE and Aβ between the isoforms.

As a more informative method of determining the degree of interaction, FLIM analysis was applied to the data. The advantage of using FLIM-FRET is that both the proportion of epitopes that are interacting and the closeness of that interaction can be independently calculated from the same data set [26], allowing us to address both the question of propensity to bind and closeness of interaction, in a well separated fashion. Three epitopes were labeled in FRET pairs (figure 1b).

Difference in Aβ-ApoE distance for each isoform (figure 1c) reached trend levels in the initial Bonferroni-Dunn post-hoc tests (p<0.1). If we examine the data by terminal, we see that Aβ-ApoE NT distance is less for ApoE3 than ApoE4 in the (p<0.05), but this is not the case for Aβ-ApoE CT (p = 0.125). These data suggest that the CT of both ApoE3 and ApoE4 interact with Aβ in senile plaques in a similar fashion, but that the NT domains adopt an isoform specific tertiary structure.

ApoE CT is closer to Aβ than ApoE NT for both isoforms. (p<0.0001: *post-hoc* test split by isoform; p<0.0005, -ApoE3-, p<0.0001, -ApoE4-). These data support a model, based on *in vitro* data, that Aβ interacts with the amphipathic domain in the C-terminal of ApoE [9], [10].

Comparisons of the interacting fractions (figure 1d) of Aβ-ApoE3 and Aβ-ApoE4 for both termini of ApoE show that a larger number of the Aβ molecules interact with ApoE4 than with ApoE3 (p<0.0001: *post-hoc* split by termini; p<0.05, -ApoE CT-, p<0.05, -ApoE NT-). Surprisingly, we also observed a clear and significant difference in the interacting fraction of Aβ-ApoE CT compared to Aβ-ApoE NT (p<0.0001, *post-hoc* split by isoform; p<0.01, -ApoE3-, p<0.01, -ApoE4-), raising the intriguing possibility that some Aβ peptides are bound to isolated ApoE C-terminal fragments (CTFs) or that some N-terminal domains become hidden from access by the reagents.

To control for the effect of autofluorescence, plaques stained only with donor fluorophore were subjected to the same analysis; no significant FLIM signal was observed. While comparison to plaques in which the acceptor fluorophore is used to stain a non-interacting, yet co-localized protein would be ideal, there is no settled candidate protein for this, according to the literature. However, we can state that this technique, including choice of filters and fluorophores is a long standing technique that may be considered validated. [28]

To directly test the model that ApoE CT binds plaques, and that ApoE NT are differentially positioned in ApoE3 and ApoE4, we performed FLIM-FRET experiments comparing the distance between the two terminal domains of ApoE associated with plaques. ApoE CT and ApoE NT were immunolabeled with donor and acceptor fluorophore respectively (figure 1e). A significant (p<0.05) but small difference in inter-terminal distance suggests that the N and C termini of ApoE decorating a plaque are quite close to one another, but that ApoE4 isoform differs from ApoE3 in that the N and C termini are slightly closer together.

In addition to this subtle difference in C-N termini distance, we observe that the N and C termini have a much higher interacting fraction for ApoE3 than ApoE4 (p<0.0001) (figure 1f). This result is consistent with the isoform specific difference in interacting fraction observed for ApoE-Aβ interactions (figure 1a), and strongly supports the hypothesis that the N-terminal domain of ApoE4 is either hidden or missing in some ApoE molecules associated with plaques.

**Figure 2:**
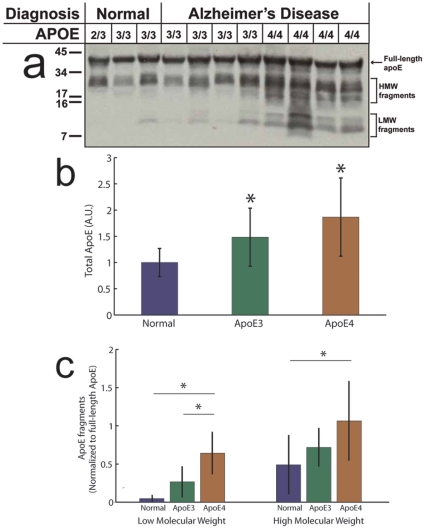
Western blots of brain homogenates from AD patients and normal aged brains. a) Using poly-clonal antibody, the distribution of ApoE fragments is clearly different between healthy aged brains and AD brains with further marked differences between genotypes. b) Comparisons of densitometry show significant increases in the amount of ApoE in Alzheimer brains, with the greatest amount in individuals homozygous for ApoE4. c) We also measured an increase in LMW (7–10 kDa) fragments in the case of AD, the presence of APOEε4 further amplifies the effect. The effect is similar but less subtle for HMW (17–34 kDa) fragments. In all cases asterisks indicate significance as assessed using ANOVA and Bonferonni-Dunn post-hoc test.

To examine whether the observed differences between ApoE3 and ApoE4 were the result of differences in ApoE cleavage in the brain, or if they are local effects associated with the plaques themselves, brain samples, taken from the Massachusetts Alzheimer Disease Research Center brain bank were homogenized and ApoE was characterized using western blot. Samples were selected to be confirmed AD and homozygous for either APOEε3 or APOEε4; non-AD control brains (homozygous for APOEε3) were also sampled. The antibody used was a commercial polyclonal goat anti-ApoE (Calbiochem, catalog #179478). Figure 2a shows an exposure that illustrates the differential banding patterns between genotypes. Densitometry data, which was obtained from a lower exposure than that shown, were averaged over 3 blots with ‘n’s of 9, 13, and 11 (cognitively normal, AD 3/3, and AD 4/4). The total concentration of ApoE was found to be significantly elevated in AD patients with APOEε3/3 genotype (p<0.001) and APOEε4/4 genotype (p<0.0001 figure 2b).

To investigate degradation and fragmentation, two ranges of interest of molecular weights were defined. The low molecular weight (LMW) band between 7–10 kDa; appears among AD patients with both APOEε3/3 and APOEε4/4, and doesn't seem to be present in normal brains. A strong band of fragments between about 17 and 34 kDa, designated High Molecular Weight (HMW), is present in all brains but is stronger and broader in AD, and particularly so in ApoE4, AD brains. Densitometry (figure 2c) reveals significant differences in the amount of LMW fragments between normal and AD(4/4) brains and between AD(3/3) and AD(4/4) brains, with the difference between normal and AD(3/3) narrowly missing significance, (3 way ANOVA P<0.0001, Bonferonni-Dunn post-hoc test: control vs AD(3/3), p<0.05; control vs AD(4/4), p<0.0001; AD(3/3) vs AD(4/4), p<0.0001). The differences in HMW fragment density were more subtle with significance detected only between normal and 4/4 (p<0.01).

**Figure 3:**
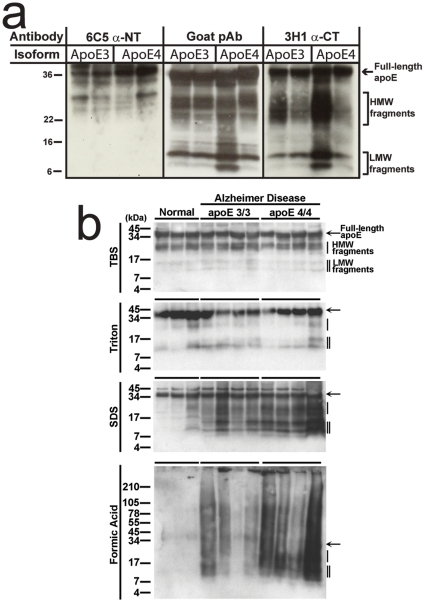
Western blots probing for the termini of ApoE. a) Blots using terminal specific antibodies reveal that the LMW band, and the lower portion of the HMW band are almost exclusively composed of C-terminal fragments. b) Blots of fractional brain extracts show that some higher molecular weight fragments exist in the TBS fraction. The Triton fraction contains comparatively few fragments of any size. The bulk of the degraded ApoE, particularly the LMW band appears in the SDS and formic acid fractions, implying that the degraded C-terminal fragments of ApoE are associated with amyloid plaques.

To separate CTFs and NTFs of ApoE, new samples were prepared from brains with the greatest proportion of fragments for both ApoE3 and ApoE4. These samples were probed with terminal specific antibodies 3H1 and 6C5 (figure 3a). Results were similar across genotypes in terms of antibody specificity for fragments. HMW fragments were detected with the anti-CTF and anti-NTF antibodies, whereas LMW fragments were detected only with anti-CT antibody. Therefore, the LMW fraction can be considered to be primarily CTF, whereas the HMW fraction is a mixture of fragments from both terminal domains.

To discover if the ApoE fragments were soluble in the brain or associated with aggregated protein, samples were sequentially homogenized in 5 volumes of pure TBS with protease inhibitor cocktail, 2% Triton X-100, and 2% SDS, the SDS insoluble pellets were sonicated in 70% formic acid (see methods). Blots of these protein fractions (figure 3b) show that there are limited numbers of HMW soluble ApoE fragments in both APOEε3/3 and APOEε4/4 brains. Very few fragments were observed in the Triton fraction. Almost all of the LMW CT fragments were found in the SDS fraction, confirming that these ApoE fragments are insoluble and therefore likely to be at least partially aggregated or associated with senile plaques.

Taken together, these data suggest that ApoE undergoes isoform specific differential cleavage in the AD brain with significantly more degradation, particularly of the CTF, for ApoE4 than ApoE3. We can also say that, consistent with our FLIM data, isolated CTFs are associated with senile plaques.

## Discussion

Inheritance of APOEε4 is known to be associated with increased risk for AD compared to the more common APOEε3. The proteins ApoE3 and ApoE4 are known to differ in the periphery where they associate with low and high density lipids respectively [29], likely because they adopt different conformations. In the brain, however, there is only one class of ApoE containing lipoprotein particle, an HDL-like particle; ApoE is also associated with Aβ deposits in senile plaques. Whether or not ApoE3 and ApoE4 present different conformations when associated with senile plaques is unknown. We utilized a new application of FRET-FLIM to interrogate ApoE-Aβ interactions, and ApoE conformation when associated with plaques in the Alzheimer brain.

**Figure 4:**
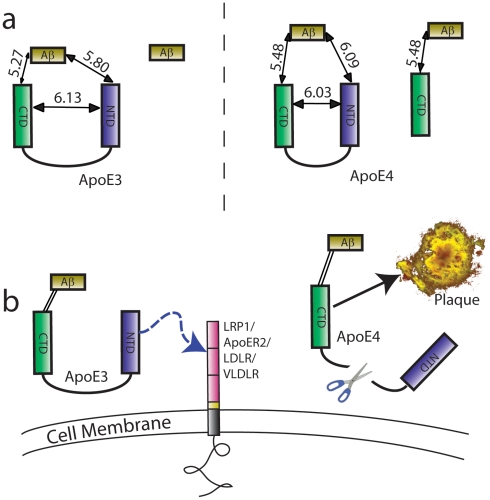
Summary of findings. a) The FLIM-FRET data can be translated into real distances by multiplying by the Forster radius of A488 and Alexafluor 555 (7 nm). The structural differences between isoforms and interactions with Aβ are compared (not to scale but distances marked in nm). The Aβ-ApoE4 distance is larger than Aβ-ApoE3 despite the fact that the propensity for interaction is greater. ApoE4 shows a tighter inter-terminal interaction although the difference is not large. This result suggests that under these conditions ApoE conformation is influenced but not dominated the domain interaction. Nevertheless, this small conformational difference could lead to a differential vulnerability to cleavage at the hinge region leading to a larger number of orphan ApoE4 C-terminal fragments bound to Aβ. b) Our proposed model for the increased risk of senile plaque associated with ApoE4. Under normal conditions, ApoE acts as a bridging protein between Aβ and one of the lipoprotein receptor proteins, thereby mediating clearance across the cell membrane. In the Case of ApoE4, the conformational difference leads to enhanced vulnerability to cleavage, which in turn leads to a loss of clearance function and enhanced deposition.

We show that 1. Both isoforms of ApoE interact closely with fibrillar Aβ, but that 2. ApoE3 interacts more closely with Aβ than ApoE4. 3. For both isoforms, the amphipathic C-terminal interacts most closely with Aβ. 4. A greater number of Aβ molecules interact with ApoE4 than with ApoE3. 5. ApoE4 adopts a conformation with slightly shorter CT-NT distance than ApoE3. 6. Fewer CTs interact with NTs in the case of ApoE4, indicating a greater level of degradation. 7. Greater amounts of ApoE can be found in Alzheimer brain than normal brains and that the effect is most pronounced among APOEε4/4 individuals. 8. In the AD brain ApoE undergoes significantly more cleavage than in normal brains, and the effect is further amplified amongst carriers of APOEε4. 9. Isolated CTFs are associated with senile plaques especially for ApoE4. The major findings of this work are summarized in a schematic form in figure 4, with the distances translated into approximate nm units. For this calculation, the Forster radius of A488 and Cy3 was approximated using the value for A488, and Alexafluor 555 (70 nm) given on the Invitrogen website, which has an almost identical excitation spectrum to Cy3.

FRET has become an established technique for assessing protein interactions and changes in protein conformation in living cells and in stained tissue [30]. While it is sometimes said that the distance over which interactions are measured in FRET are too great to prove molecular interactions, it is also true that FRET does not measure the distance between nuclei of bound atoms, rather the distances measured here, of the order of 5–10 nm, are entirely reasonable for the detection of distances between two epitopes on large proteins. It is also the case that while for single molecule FRET it is conceivable that the FRET signal is due to happenstance proximity between two molecules, in a real system, with many molecules, such statistical anomalies are rare and would not create a significant signal. This point was elegantly demonstrated in work by Server et al [28], by comparing a colocalization and FLIM measurement of dynamin-auxilin interaction for both wild-type and a mutated dynamin whereby the interaction was disrupted without altering the spatial distribution of the proteins. It is worth noting however, that this technique does not tell us about the nature of the interaction, these molecules may interact either by hydrogen bonding or purely through hydrostatic bonding. This uncertainty, however, does not change the interpretation of the data.

These data directly test the hypothesis that an isoform specific difference in ApoE-Aβ interaction can be observed in fibrillar Aβ deposits. Natively lipidated ApoE3 has been shown to have a greater binding affinity to Aβ than ApoE4 [31]. We confirm that of the two ApoE domains, the ApoE CT is in closer apposition to Aβ, supporting the notion that the C-terminal domain, containing the major lipid binding region is the region that interacts with Aβ, and that the CT of both ApoE3 and ApoE4 interact closely with Aβ. However, the difference between the two distances is small enough to suggest that ApoE might encapsulate Aβ, in a similar fashion to the way in which it interacts with lipids [32].

There are however, distinct differences in the nature of the interaction. Specifically, ApoE3 interacts more closely with Aβ in senile plaques. In addition, a greater number of Aβ molecules interact with ApoE4 than ApoE3. Finally, the ApoE N-terminus appears to be buried or missing, especially in ApoE4 cases; biochemical analyses suggest that ApoE undergoes cleavage, again especially for ApoE4, suggesting that the N-terminus is indeed separated from the C-terminus in ApoE4 plaques. This interpretation is consistent with the previous studies which indicate that ApoE4 undergoes more degradation in the AD brain than ApoE3 [33].

There is a well documented conformational difference between ApoE3 and ApoE4 caused by an interaction between the two highly structured terminal domains that is stronger in the case of ApoE4 [4], [34]. One consequence of the isoform specific domain interaction and conformational change may be the relative vulnerability to cleavage of the unstructured hinge region. If this were the case, then we postulated that we might detect a differential likelihood in observing N-C terminal interactions. Our analysis directly tested this hypothesis and revealed that ApoE4 adopted a slightly tighter conformation with an increased closeness of N-C terminal interaction. It is surprising that the conformational difference between the isoforms was comparatively small compared with expectations based on *in vitro* data [34], [35], [36]. It is worth noting that the effects of ApoE isoform take many decades to cause an effect in patients, a more drastic effect might be expected to result in onset of symptoms far earlier, as is observed in familial AD cases. This result particularly highlights the need for *in situ* measurements of endogenous proteins to confirm that observations made in reduced preparations, like tissue culture or in solution, can be extended to the native environment.

We also note a greater interacting fraction between the Aβ and ApoE CT, compared with ApoE NT. At the same time, the lower interacting fraction between the NT and CT of ApoE, especially for ApoE4, indicates that either the epitope for the N-terminal specific ApoE antibody has been partially occluded or an increased number of isolated C-terminal fragments exist in senile plaques in the case of ApoE4. Since previous biochemical studies suggest that ApoE4 is cleaved at the hinge region to a greater extent than ApoE3 [21], [37], and that elevated levels of ApoE4 CTFs are found in the brains and cerebral spinal fluids of carriers of APOEε4 [37], we tested the possibility that the decreased interacting fraction that we observed indicates a pathologically elevated cleavage of ApoE4. Work by Riddell and Zhou [38] in mice suggests that enhanced degradation of ApoE4 by astrocytes leads to lower levels of ApoE and thereby impacts clearance, while recent work from the Garner lab [39] suggests that ApoE3 suffers greater fragmentation without regards for whether the patient had AD as assessed in the TBS fraction of human brain. By contrast, we find increased cleavage in AD, and especially, in ApoE4 individuals, using both biochemical assays and, importantly, FRET based assays of the protein *in situ*. Since ApoE contains an unstructured region and is somewhat prone to aggregation, it is possible that differences in antibody choice and specificity may play some role in the differences between observations.

The question remains as to how these observations shape our understanding as to the differential role of these two isoforms of ApoE on Aβ clearance and deposition. We must consider how these data fit with the observation that ApoE4 is associated with greater Aβ deposition or clearance of Aβ from the neuropil. One set of hypotheses states that Aβ is cleared through the blood brain barrier to the periphery and ApoE is thought to impede that process [40], perhaps in an isoform dependant manner. In addition, ApoE may mediate proteolytic degradation through neurons, astrocytes or microglial cells [41] by acting as a bridging protein between Aβ and one or more of several candidate receptors, particularly LRP1 [14] or ApoER2 [42].

The presence of isolated ApoE CT fragments may also contribute to plaque deposition or stabilization. Our data do not directly assess ApoE's role in clearance; instead, however, the current data demonstrate that ApoE3 and ApoE4 differentially interact with fibrillar Aβ deposits.

These data, taken together lend strong support to the hypothesis that there is an isoform specific mechanistic effect on plaque deposition and clearance that is implicated in the positive correlation between ApoE4 dose and plaque density [13], [14] and strengthens the case for therapeutic intervention targeted at ApoE4's unique tertiary structure [43].

## Materials and Methods

### Tissue and immunostaining

Massachusetts Alzheimer Disease Research Center (ADRC) Brain Bank provided free floating human brain sections (50 µm) from patients homozygous for APOEε3 or APOEε4 and with postmortem confirmed AD.

All sections were pretreated with 10 mM citrate buffer pH6 for 10 minutes at 95°C before immunostaining. Sections were incubated with either C-terminal specific ApoE antibody, 3H1 (aa 243-272, 1∶100, Ottawa Heart Institute); or 3D6, which is directed against Aβ (1∶500, Elan Pharmaceuticals) antibodies and visualized with secondary antibody conjugated to Alexafluor 488 (A488) (Invitrogen). After sequential washing, the sections were incubated with 6C5 antibody (ApoE NT, aa 1-15, 1∶1000) (Ottawa Heart Institute) directly conjugated to Cyanine 3 (Cy3) (GE Healthcare). To probe the interaction between Aβ and ApoE CT; Aβ was labeled as above and ApoE CT was labeled using 3H1, directly conjugated to Cy3 [44].

### Fluorescence Lifetime Microscopy

Sections were imaged using a Zeiss LSM-510 microscope, which has excitation and emission channels for confocal, near infrared and FLIM imaging. Cortical neuritic senile plaques were initially located using visual inspection of both fluorophores under epifluorescent wide field microscopy. Upon identification of a plaque, multi-track confocal images of A488 (green) and Cy3 (red) were obtained to confirm the co-localization of the two fluorophores, followed by FLIM imaging. Excitation of A488 for FLIM was achieved with a picosecond 2-photon laser (Tsunami, Spectra-Physics). Images were taken with the laser tuned to 760 nm, which we have found to be the most efficient two photon wavelength for selective excitation of A488 while minimizing autofluorescent contribution.

Analysis was performed using a combination of SPCImage v2.9.5 (Becker and Hickl, GmbH) and previously described in house post analysis programs; *χ^2^ filter* and MUGLE [27], written in MATLAB (Mathworks, MA, USA). In brief, the analysis method is a multi-stage process. In the first instance, all images were fit with mono-exponential functions in SPCImage. Matrices of brightness, lifetime, and goodness of fit parameter (χ_r_^2^), are imported into the program chifilt, in which χ_r_^2^ is used as a pseudo-contrast for autofluorescence. A cutoff for χ_r_^2^ is selected by comparing all images; binary masks are created and saved. A baseline fluorescent lifetime for the donor fluorophore is obtained by analyzing the data from sections stained only with donor and used as a fixed prior lifetime for bi-exponential fits of sections stained with both donor and acceptor. During both of these later two stages, pixels are discarded by both the use of a region of interest around the plaque, and the masks created in the first stage of analysis. The intensity weighted histogram of the lifetimes is fit to one or more Gaussians using MUGLE.

Differences in interacting fraction; A_F_/(A_F_+A_NF_), where A is the pre-exponential factor and the subscripts F and NF refer to the FRET quenched and unquenched components were calculated along with normalized inter-terminal distance based on FRET efficiency
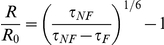
where R_0_ is the Förster radius, at which 50% of energy is transferred from donor to acceptor [26]. Results were analyzed for significance by analysis of variance (ANOVA) and Bonferoni-Dunn *post hoc* tests.

### Preparation of Brain extracts

Cortical gray matter from the temporal lobe of AD and non-demented control patients was homogenized in RIPA lysis buffer (25 mM Tris-HCl pH 7.6, 150 mM NaCl, 1% NP-40, 1% sodium deoxycholate, 0.1% SDS) containing protease inhibitor cocktail (Roche, cat #11836153001), and then subjected to centrifugation at 16,000×g for 20 min at 4°C. The supernatant was used for SDS-PAGE and Western blot. For fractional brain extracts, samples were sequentially homogenized in 5 volumes of; TBSI (Tris-buffered saline containing a protease inhibitor cocktail [Roche]), 2% Triton X-100, and 2% SDS, with 25 strokes on a mechanical Dounce homogenizer, and subjected to centrifugation at 260,000×g for 20 min at 4 degrees for each fractional extract. In each case, the supernatant was drawn off and used for Western blot analysis. For the final extraction, SDS insoluble pellets were sonicated in 70% formic acid, centrifuged at 260,000×g for 30 minutes at 4 degrees. The supernatant was evaporated and resolubilized in dimethyl dulfoxide (DMSO) for Western blot analysis.

### SDS-PAGE and Western Blot

Protein concentrations were determined by BCA assay. Equal amounts of total protein were loaded per well and electrophoresed through 10–20% Tricine or Tris-Glycine gradient gels (Invitrogen, catalog #E66255BOX and #EE61355BOX) and then transferred to PVDF membrane (PerkinElmer, catalog #NEF1002001). The membranes were incubated in blocking buffer (5% nonfat dried milk in TBS containing 0.01% Tween-20) for 1 h at room temperature, and then incubated with primary antibody diluted in blocking buffer for 1–2 h at room temperature. Primary antibodies used were: goat anti-ApoE (Calbiochem, catalog #179478), 3H1 mouse anti-C-terminal ApoE (Ottawa Heart Institute), and 6C5 mouse anti-N-terminal ApoE (Ottawa Heart Institute). Membranes were then incubated with HRP-conjugated horse anti-goat (Vector, catalog #PI-9500) or goat anti-mouse (Bio-Rad, catalog #170-6516) secondary antibody diluted in blocking buffer for 1 h at room temperature and protein detected by enhanced chemiluminescence (PerkinElmer, catalog #NEL102001).
